# Systemic inflammation alters central 5-HT function as determined by pharmacological MRI

**DOI:** 10.1016/j.neuroimage.2013.02.046

**Published:** 2013-07-15

**Authors:** Yvonne Couch, Chris J. Martin, Clare Howarth, Josie Raley, Alexandre A. Khrapitchev, Michael Stratford, Trevor Sharp, Nicola R. Sibson, Daniel C. Anthony

**Affiliations:** a Department of Pharmacology, University of Oxford, Mansfield Rd, Oxford, OX1 3QT, UK; b CR-UK/MRC Gray Institute for Radiation Oncology and Biology, Department of Oncology, University of Oxford, Churchill Hospital, Oxford, OX3 7LJ, UK

**Keywords:** Serotonin, Inflammation, BOLD fMRI, 5-HT_2A_ receptor, Rat

## Abstract

Considerable evidence indicates a link between systemic inflammation and central 5-HT function. This study used pharmacological magnetic resonance imaging (phMRI) to study the effects of systemic inflammatory events on central 5-HT function. Changes in blood oxygenation level dependent (BOLD) contrast were detected in selected brain regions of anaesthetised rats in response to intravenous administration of the 5-HT-releasing agent, fenfluramine (10 mg/kg). Further groups of rats were pre-treated with the bacterial lipopolysaccharide (LPS; 0.5 mg/kg), to induce systemic inflammation, or the selective 5-HT_2A_ receptor antagonist MDL100907 prior to fenfluramine. The resultant phMRI data were investigated further through measurements of cortical 5-HT release (microdialysis), and vascular responsivity, as well as a more thorough investigation of the role of the 5-HT_2A_ receptor in sickness behaviour. Fenfluramine evoked a positive BOLD response in the motor cortex (+ 15.9 ± 2%) and a negative BOLD response in the dorsal raphe nucleus (− 9.9 ± 4.2%) and nucleus accumbens (− 7.7 ± 5.3%). In all regions, BOLD responses to fenfluramine were significantly attenuated by pre-treatment with LPS (p < 0.0001), but neurovascular coupling remained intact, and fenfluramine-evoked 5-HT release was not affected. However, increased expression of the 5-HT_2A_ receptor mRNA and decreased 5-HT_2A_-dependent behaviour (wet-dog shakes) was a feature of the LPS treatment and may underpin the altered phMRI signal. MDL100907 (0.5 mg/kg), 5-HT_2A_ antagonist, significantly reduced the BOLD responses to fenfluramine in all three regions (p < 0.0001) in a similar manner to LPS. Together these results suggest that systemic inflammation decreases brain 5-HT activity as assessed by phMRI. However, these effects do not appear to be mediated by changes in 5-HT release, but are associated with changes in 5-HT_2A_-receptor-mediated downstream signalling pathways.

## Highlights

• Sickness has significant effects on 5-HT mediated phMRI.
• These effects are not caused by changes in 5-HT-release mechanisms.
• These effects are not caused by neurovascular uncoupling.
• LPS-induced changes in phMRI may reflect 5-HT_2A_-mediated signalling cascades.
• 5-HT_2A_-mediated behaviours are significantly changed by sickness.

## Introduction

Evidence indicates that abnormalities in brain 5-HT function, responses to citalopram for example, persist in patients who are clinically recovered from depression, but at risk of relapse (Bhagwagar et al., 2002). Understanding biological variation in the 5-HT system caused by key risk factors is central to the search for effective antidepressant treatment. Inflammation is thought to be an important risk factor in the aetiology of depression (Hughes et al., 2012). Furthermore, anti-depressant drugs are known to reverse inflammation-induced depression, suggesting that cytokine production may directly affect the 5-HT system. For example, therapy with the cytokine interferon-α results in 25% of patients suffering from a depressive episode as an adverse effect which is subsequently managed therapeutically by 5-HT targeted antidepressants (Horikawa et al., 2003). Similarly, potent broad-spectrum inflammogens, such as the bacterial endotoxin lipopolysaccharide (LPS) and attenuated *Mycobacterium bovis*, have been shown to induce depressive-like symptoms, often known as sickness behaviours, in rodents and induce changes in the 5-HT system (O'Connor et al., 2009). Sickness behaviours are defined behavioural adaptations in response to an invading pathogen and are observed in both human patients and in rodents (Hart, 1988). They are now thought to arise from an interaction between the immune system and the 5-HT system (O'Connor et al., 2009). This interaction between 5-HT and the immune system is bidirectional in nature — clinically depressed patients with no obvious systemic infection often present with high levels of circulating cytokines and reduced tryptophan levels (Alesci et al., 2005; Levine et al., 1999). Reduced tryptophan levels indicate a potential link between the systemic immune system and the central 5-HT system, however, the mechanisms by which this link is established remain unclear.

The mechanisms by which inflammatory molecules affect 5-HT, its signalling pathways, and subsequent mood, are currently under debate. Dantzer and colleagues have suggested that proinflammatory stimuli, such LPS or interleukin-1β (IL-1β), increase activity in the tryptophan metabolising enzyme indoleamine 2,3-dioxygenase (IDO), resulting in lowered tryptophan availability and therefore decreased 5-HT synthesis (O'Connor et al., 2009). However, data currently available is contradictory. Linthorst and colleagues demonstrated increased 5-HT levels in hippocampal dialysates following systemic administration of LPS or intraventricular administration of IL-1β (Linthorst et al., 1995), whilst Wilkinson et al. failed to demonstrate any functional link between systemic infection and central 5-HT levels (Wilkinson et al., 1991). Decreased presynaptic 5-HT function as a mechanism for sickness behaviours would be consistent with other findings, including evidence that LPS and other proinflammatory stimuli increase expression of the 5-HT transporter (Ramamoorthy et al., 1995; Zhu et al., 2010), which would reduce 5-HT availability in the synapse (Jennings et al., 2006).

*In vivo* functional magnetic resonance imaging (fMRI) in combination with 5-HT-targeted pharmacological challenges, termed pharmacological MRI (phMRI), offers a translational non-invasive neuroimaging approach to model 5-HT function that is complementary to measurements of 5-HT receptor levels offered by techniques such as positron emission tomography (Moresco et al., 2006). phMRI utilises a haemodynamic correlate of neural activity (the blood oxygenation level dependent [BOLD] signal), which is considered an aggregate index of excitation–inhibition activity resulting from afferent neuromodulatory input to a given brain structure (Logothetis, 2008). Ourselves, and others, have previously demonstrated in preclinical models that it is possible to monitor 5-HT function using phMRI, with BOLD signals being sensitive to drug-evoked increases in endogenous 5-HT levels and 5-HT receptor-specific modulations of neuronal function (Rauch et al., 2008; Preece et al., 2009). Moreover, clinical studies have demonstrated that 5-HT augmentation through 5-HT reuptake blockade (Anderson et al., 2008; Murphy et al., 2009) evokes region-specific alterations in BOLD responses both under baseline conditions and during emotional processing tasks. Although the 5-HT receptor subtypes underpinning 5-HT drug-evoked increases in BOLD are not yet known, our previous studies demonstrated that fenfluramine-evoked changes in activity-dependent gene expression involved 5-HT_2A_ receptor activation (Hirani et al., 2003).

Taken together, the above studies reinforce the concept that it is possible to measure the functional effects of 5-HT modulation using phMRI. The primary aim of this study, therefore, was to use phMRI, in combination with microdialysis, behavioural, and molecular biology approaches, to determine the effects of systemic inflammation on central 5-HT function.

## Materials & methods

### Animal preparation

Male Sprague–Dawley rats (n = 5 per group, 250–300 g; Harlan, UK) were anesthetised with 2–3% halothane in a 60:40 mixture of N_2_O:O_2_. Subsequently, halothane was reduced to 1.5–2.0% and the animals were tracheotomised and artificially ventilated. Core body temperature was regulated and maintained at 37 °C throughout the experiment, using both heating and cooling systems within the magnet. The left femoral artery and vein were cannulated for measurement of blood pressure, withdrawal of blood samples and to enable i.v. administration of drugs or vehicle (0.9% saline). All drug doses were calculated to maintain injection volumes of 0.1 ml, irrespective of administration route. Throughout data collection, 0.8–1.0% halothane was used as maintenance anaesthesia. All experiments were approved by the UK Home Office Animals (Scientific Procedures) Act (1986) in line with EU directives on animal experimentation. All experiments were further approved by local ethical committees in line with the 3Rs principles.

### Magnetic resonance imaging

Preliminary dose–response studies using this methodology showed significant activation after administration of fenfluramine in the motor cortex, nucleus accumbens and dorsal raphe (data not shown), therefore these regions have been focussed on in this study. Animals (n = 5–6/group as specified in the text) were placed in a quadrature birdcage radiofrequency coil (internal diameter 5 cm) with an integral stereotaxic frame. Electrocardiogram (ECG) was monitored throughout and body temperature maintained at 37 °C. MRI data were acquired using a 7 T magnet (Magnex Scientific, Abingdon, UK), and a Varian Inova spectrometer (Varian Inc, Palo Alto, USA). Sets of five coronal images spanning the rat forebrain were acquired using a T2*-weighted FLASH sequence (TE = 25 ms, TR = 500 ms, 40° flip angle, field of view 80 × 40 mm, matrix size 256 × 128, slice thickness 1.5 mm). Dynamic updating of first order shims was used for each slice to maximise fMRI sensitivity (Blamire et al., 1996; Koch et al., 2006). Each set of five images was acquired over a 2 min time period. Anatomical images of the same five coronal slices were acquired using a T2-weighted fast spin echo sequence (TR = 3 s, TE = 45 ms, field of view 30 × 30 mm, matrix size 128 × 128). Images were acquired continuously throughout the experimental protocol, which consisted of 15 min of baseline imaging, followed by a bolus i.v. administration of either fenfluramine (10 mg/kg i.v.) or vehicle over 1 min, and a further 85 min of image acquisition. Thus, the first 16 min (8 images) of data acquisition are considered as baseline.

To determine the effect of systemic inflammation on the phMRI response to fenfluramine, animals were injected intraperitoneally with 0.5 mg/kg LPS (*E. coli* strain O111:B4; Sigma-Aldrich) in sterile pyrogen-free saline to induce a systemic inflammatory response 6h prior to fenfluramine administration (n = 6). Previous data show that 0.5 mg/kg LPS is required to produce a robust febrile response (Derijk et al., 1993). Control animals (n = 5) were injected with vehicle, instead of LPS, 6 h prior to fenfluramine administration. Two further control groups were included that received either LPS (n = 3) or saline (n = 4) 6 h prior to phMRI during which vehicle was injected in place of fenfluramine. The four groups are denoted as follows: LPS-Fen, Veh-Fen, LPS-Veh and Veh-Veh.

In a further group of animals the effects of blocking the 5-HT_2A_ receptor on the phMRI response to fenfluramine were assessed. In these animals, a single dose of MDL100907 (0.5 mg/kg) was administered i.p. *via* an indwelling cannula 15 min prior to either fenfluramine (n = 6) or vehicle (n = 3) administration. The MRI acquisition was started 15 min prior to MDL100907 administration and a further 15 min of baseline data were acquired following MDL100907 injection and prior to fenfluramine/vehicle administration (total baseline data = 30 min). In these groups image acquisition post-fenfluramine (or vehicle) was shortened to 75 min owing to the longer baseline period. The data were compared to animals injected with either fenfluramine (n = 5) or vehicle alone (n = 4). These animals were not pre-injected with vehicle given that the previous experiment had demonstrated no effect of saline administration on the BOLD signal and, hence, the baseline period was only 15 min in duration. Thus, the data from this experiment are shown from 15 min prior to either fenfluramine or vehicle injection for all groups. Again, the baseline period for analysis was taken as 16 min (8 images) in total to allow for the fenfluramine injection.

### MRI data analysis

Analysis of the fMRI datasets was carried out using AFNI (http://afni.nimh.nih.gov/afni/) and FEAT (http://www.fmrib.ox.ac.uk) software packages (Jenkinson et al., 2012; Woolrich et al., 2009; Smith et al., 2004). Datasets were corrected for any motion artefacts using AFNI, and spatial smoothing performed in FEAT using a Gaussian kernel of FWHM 1 mm. Statistical analysis was performed using a general linear model within FEAT. The design matrix consisted of a simple temporal model of the expected response, with 0 specifying the baseline period and 1 or − 1 specifying signal increases or decreases during the post-drug period respectively. To generate activation maps, we used the cluster-based thresholding approach described in Smith et al. (2004). Images were converted to Z-statistics and thresholded to identify voxel clusters. A Z-statistic threshold of Z = 3.51 was used to define contiguous clusters and the significance level of each clusters (from Gaussian random field theory, Friston et al., 1995) was compared with a cluster probability threshold of p < 0.05. A colour-coded z-statistic map was then overlaid onto the corresponding phMRI gradient echo image. With reference to the anatomical images and a stereotaxic atlas (Paxinos et al., 1980), regions of interest (ROI) were manually defined on the functional datasets for anterior motor cortex, nucleus accumbens and dorsal raphe nucleus. The average BOLD signal intensity changes across all pixels over time within these ROIs was determined, expressed as percentage change from baseline signal intensity, and plotted as a function of time.

### Brain microdialysis

Microdialysis probes were fabricated in-house using stainless steel cannulae (23G, Cooper's Needle Works Ltd, United Kingdom) with a semi-permeable membrane tip (3 mm effective tip length; 200 mm I.D., 40,000 MW cut-off, 60 Å pore size, Hospal AN 69). Animals were prepared as described above (n = 5 or 6 per group). Following tracheotomy and vessel cannulations, animals were placed in a stereotaxic frame (Stoetling Co., USA), the skull was exposed and a burr-hole drilled above the right anterior motor cortex and the microdialysis probe inserted stereotaxically (A/P + 2.2 mm; M/L + 2.8 mm; D/V − 1 mm). The microdialysis probe was perfused with artificial cerebral spinal fluid (aCSF; 140 mM NaCl, 3 mM KCl, 1.2 mM Na_2_HPO_4_, 0.27 mM NaH_2_PO_4_, 1 mM MgCl_2_, 2.4 mM CaCl_2_ and 7.2 mM glucose) at a flow rate of 2 μl/min (CMA/100, Carnegie Medicine). The probe was perfused for 1.5–2 h *in situ* prior to the start of dialysate collection. Samples were subsequently collected over consecutive 20 min periods. At this point animals were injected with fenfluramine (10 mg/kg i.v. in 0.1 ml saline) as for the phMRI study, and a further six 20 min dialysate samples were collected.

### High pressure liquid chromatography

Dialysate samples were analysed using HPLC with electrochemical detection and separated with an ACE column (C18, 3 μm, 125 × 3 mm + ACE C18 guard, 10 × 3 mm run at 35°C). Samples were carried by an eluent (12.5% methanol, 130 mM NaH_2_PO_4_, 0.85 mM Na_2_EDTA, 0.1 mM 1-octanesulphonic acid, pH 3.55) pumped with a flow rate of 0.6 ml/min (Waters 2695 HPLC Pump). Samples were detected using a glassy carbon electrode held at + 0.75 V (Dionex ED40). The dialysate content was determined with reference to daily-calibrated standard solutions in 0.06 M perchloric acid (5 pmol 5-HT and 5-HIAA). Chromatograms were displayed and analysed using Waters Empower 2 software.

### Laser Doppler flowmetry

Six hours after either LPS or saline injection, animals (n = 5 per group) were placed in a stereotaxic frame (Kopf Instruments), and a region of skull overlying the right anterior motor cortex was thinned to translucency using a dental drill. A laser Doppler probe (Perimed Probe 403, Jarafalla, Sweden) was positioned above the visible cortical surface. Carbon fibre stimulating electrodes were inserted through burr holes made in the skull overlying the contralateral MCx and advanced over the cortical surface to a position overlying the anterior motor cortex (Austin et al., 2003). Electrical stimuli were delivered to the contralateral MCx and recordings of stimulus evoked cerebral blood flow changes were recorded using the laser Doppler probe. For each laser Doppler experimental session, 2-s stimuli of 5, 10, 20, 30 and 40 Hz were presented in a randomised sequence (10 repeats at each frequency) with a stimulus pulse width of 0.3 ms and an inter-stimulus interval (ISI) of 25 s. All stimulus presentation was controlled through a 1401 plus (CED Ltd., UK) running custom-written code.

### Autoradiography

Fresh frozen brain tissue was cut coronally at 12 μm. Adjacent tissue sections were incubated with either 2 nM [3H]Ketanserin or 2 nM [3H] with 10 μM methylsergide. Sections were then placed in a cassette with Kodak® BioMax® MR film and exposed for approximately 6 weeks. Analysis was performed using MCID-Core software.

### (±)-1-(2,5-dimethoxy-4-iodophenyl)-2-aminopropane (DOI) induced wet dog shake behaviour

Animals received a single dose of LPS as with previous experiments and were allowed to recover for *c*. 6 h. On or around the 6 hour time point animals received a single dose of DOI (1 mg/kg s.c.) and were observed for 20 min, during which the number of wet dog shakes (WDS) was counted. WDS behaviour constituted anything from a head twitch to a full body shake and data are presented as total WDS over 20 min.

### Statistics

In each case, the phMRI data were normalised to the first 16 min period of no-drug baseline acquisition (2 min temporal resolution; baseline = 8 images). The phMRI data were then analysed in two different ways: in the first instance, repeated measures analysis of variance (ANOVA) was used to compare the timecourses in each group over the first 46 min of data acquisition (16 min baseline + 30 min after drug administration); in the second case, the mean BOLD signal intensity was averaged over the first 30 min post-fenfluramine (or vehicle) administration (minutes 17–46 of acquisition) and assessed using ANOVA to identify statistical differences between the experimental groups. Where significant differences were found in the LPS study, *post-hoc* Dunnett's tests were used to identify differences between each group and the Veh-Veh group. Where significant differences were found in the MDL100907 study, post-hoc Tukey's tests were used to identify pairwise differences between the groups. CBF data were analysed by computing the area under the curve (AUC) for each animal's trial-averaged response (at each stimulation frequency). T-tests were performed at each stimulation frequency to compare data between experimental groups. Microdialysis data were analysed by t-test of the average area under curve across each group. All other data was analysed by standard Student's t-tests.

## Results

### Effect of fenfluramine on BOLD in rat brain regions

**Fig. 1:**
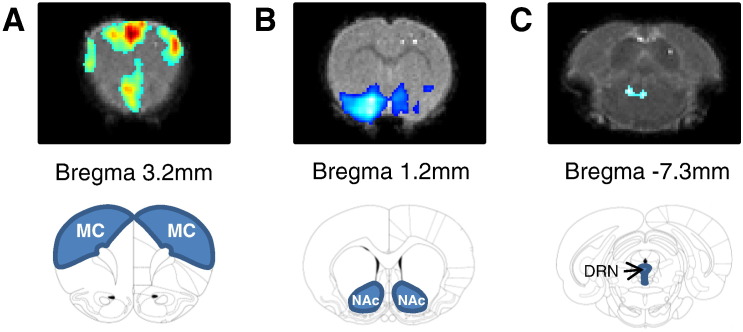
BOLD activation maps and ROIs. Representative z-score maps and Paxinos atlas images showing statistically significant BOLD responses in (A) anterior motor cortex, (B) nucleus accumbens and (C) dorsal raphe nucleus.

**Fig. 2:**
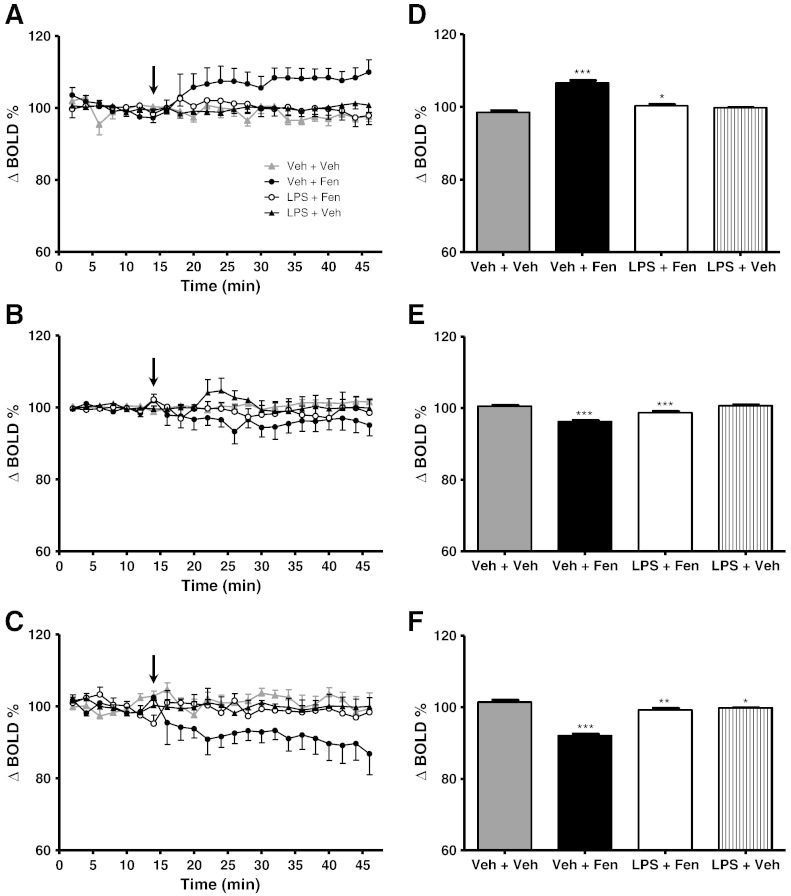
(A–C) Time course plots showing the change in BOLD signal intensity in (A) motor cortex, (B) nucleus accumbens, and (C) dorsal raphe nucleus after administration of either LPS or vehicle (0.9% saline i.p.) at t-6 h followed by either fenfluramine (10 mg/kg i.v.) or vehicle (0.9% saline i.v.) during imaging. Fenfluramine or vehicle was administered at t = 15 min (arrow). Data represent mean ± SEM (n = 6). (D–F) Bar graphs showing mean ± SEM of the signal intensity over the first 30 min after fenfluramine or vehicle administration. *p < 0.05, **p < 0.01, ***p < 0.0001 (with respect to Veh-Veh).

Physiological parameters were maintained within normal ranges throughout each experiment: body temperature = 37 ± 0.5 °C; pCO_2_ = 35–42 mm Hg; pO_2_ ≥ 90 mm Hg; pH = 7.4 ± 0.1; mean arterial blood pressure = 120 ± 20 mm Hg. Fenfluramine (10 mg/kg i.v.) evoked significant and prolonged changes in BOLD signal intensity in all three brain regions studied (Figs. 1 & 2A–C); anterior motor cortex (MCx), nucleus accumbens (NAc) and dorsal raphe nucleus (DRN). In particular, bilateral increases in BOLD signal intensity were evident in the MCx, whilst decreases in BOLD signal intensity were found in the NAc (Fig. 2B) and DRN (Fig. 2C). The maximum change observed was + 15.9 ± 2% of baseline in the MCx, − 7.7 ± 5.3% in the NAc and − 9.9 ± 4.2% in the DRN.

### Effect of systemic inflammation on fenfluramine-induced BOLD responses

Pre-treatment with LPS (0.5 mg/kg i.p.) 6 h prior to fenfluramine administration significantly reduced the BOLD response to fenfluramine. In all brain regions examined, ANOVA across the first 46 min of the time-course data (16 min baseline + 30 min post-fenfluramine or vehicle; Figs. 2A–C) showed highly significant (time p < 0.0001 F_7,414_ = 28.83, group:time p < 0.0001 F_21,414_ = 66.58) differences in the BOLD time-course response to fenfluramine between the groups. However, in the MCx and DRN, whilst *post-hoc* Dunnett's tests showed that whilst the Veh-Fen time-course was significantly different to the Veh-Veh group (MCx, DRN p < 0.0001), neither the LPS-Fen nor the LPS-Veh time-courses were significantly different to the Veh-Veh group. In the NAc both the Veh-Fen and the LPS-Fen groups were significantly different to the Veh-Veh group, albeit to differing degrees; p < 0.0001 and p < 0.05, respectively.

In the analysis of the averaged first 30 min of data acquired post-fenfluramine injection (see Materials & methods), highly significant (*post-hoc* Dunnett's test, p < 0.0001) differences were again found between the Veh-Fen and Veh-Veh groups in all regions (Figs. 2D–F). However, in this case small, but significant, differences were also found between LPS-Fen and Veh-Veh groups in all regions (*post-hoc* Dunnett's test MCx p < 0.05, NAc p < 0.0001 and DRN p < 0.01). In addition, The LPS-Veh group in the DRN showed a significant difference to the Veh-Veh group (*post-hoc* Dunnett's test p < 0.05). These data suggest some residual effects of fenfluramine, although from the time course analysis these effects appear minimal with the BOLD responses to fenfluramine being largely abolished (Figs. 2A–C).

### Effect of systemic inflammation on fenfluramine-evoked release of 5-HT

**Fig. 3:**
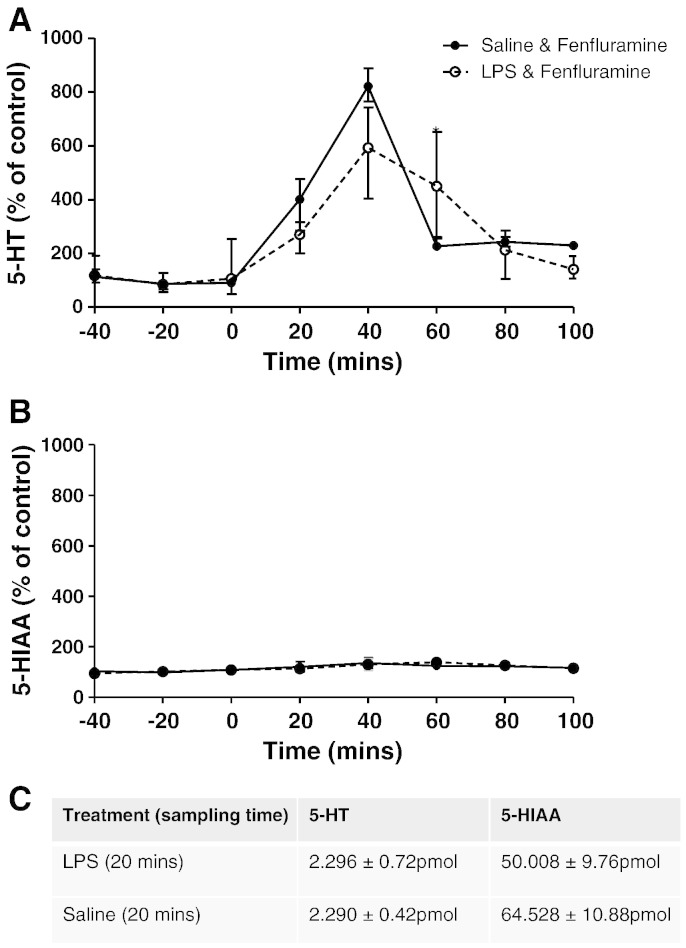
Microdialysis measurements of extracellular levels of 5-HT (A) and its main metabolite 5-HIAA (B), and the absolute concentrations of both (C), in the MCx following fenfluramine administration in control and LPS pre-treated rats. Fenfluramine (10 mg/kg i.v.) was administered at t = 0 to animals that had received an i.p. injection of saline (solid line; n = 6) or LPS (0.5 mg/kg i.p.; broken line; n = 5) 6 h prior to t = 0. Data were calculated as a percentage of baseline (mean ± SEM values are shown).

To determine whether attenuation of the fenfluramine-evoked BOLD response by LPS was due to decreased 5-HT release, *in* vivo microdialysis of the MCx was carried out under the same conditions as the phMRI experiments. The MCx was chosen for these studies based primarily on the fact that the largest BOLD changes in response to fenfluramine were found in this area. However, Manganotti et al. (2001); Herwig et al. (2002) have also shown that antidepressant treatment increases the excitability of the motor cortex, possibly *via* a 5-HT-related mechanism. Administration of fenfluramine (10 mg/kg i.v.) induced an increase in 5-HT in cortical dialysates (Fig. 3). This increase in 5-HT was greatest 40 min post-fenfluramine injection and 5-HT levels returned to baseline within the following 60 min. In comparison, fenfluramine also evoked an increase in 5-HT in animals pre-treated with LPS (0.5 mg/kg i.p., 6 h) and the magnitude 5-HT response and duration of effect was not significantly different to animals receiving fenfluramine alone (Fig. 3). Fenfluramine had no significant effect on the levels of the principal 5-HT metabolite 5HIAA, and this was not different in animals treated with LPS.

### Neurovascular coupling experiments

**Fig. 4:**
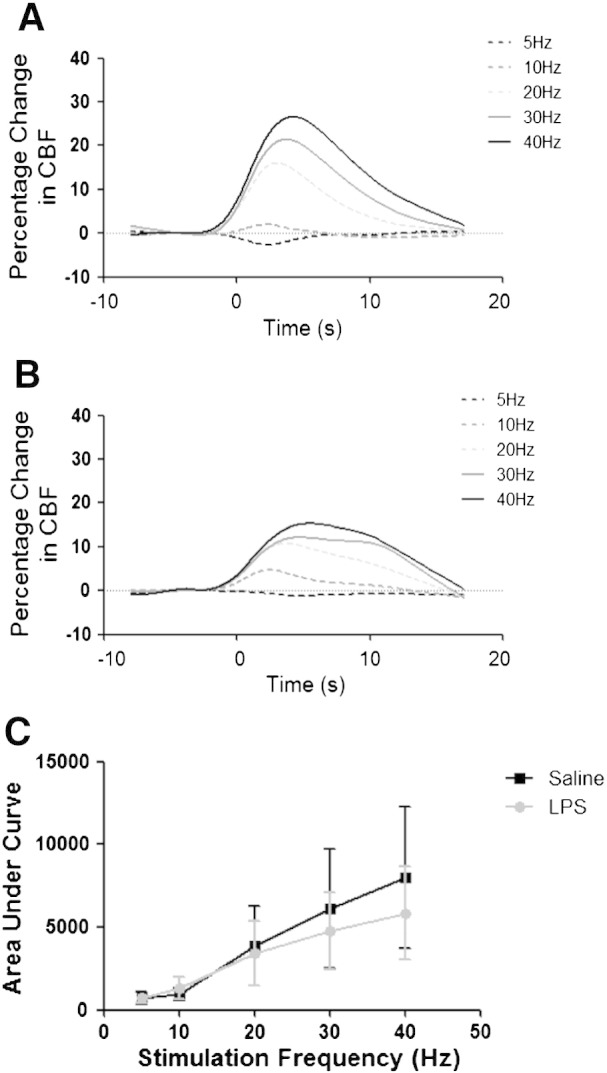
Effect of LPS treatment on neurovascular coupling and functional hyperaemia. Time course of cerebral blood flow (CBF) changes recorded in primary somatosensory MCx in response to electrical stimulation of the contralateral somatosensory MCx in (A) saline treated and (B) LPS treated animals (n = 3 per group). Stimuli were delivered using carbon fibre electrodes positioned overlying the somatosensory MCx and the stimulus evoked CBF changes were recorded using laser Doppler flowmetry probe positioned over the corresponding contralateral MCx. Stimuli consisted of a 2-s train of 0.3 ms 1.5 mA pulses at one of 5 frequencies (5, 10, 20, 30 and 40 Hz). (C) CBF responses over the range of stimulation frequencies were quantified by determining area under the curve for the mean response to each condition for each animal.

Laser-Doppler flowmetry (LDF) monitoring of stimulus-evoked cerebral blood flow (CBF) changes was made to verify that neurovascular coupling and functional hyperaemic responses remained intact post-LPS treatment (Figs. 4A–B). Six hours post-LPS administration (0.5 mg/kg i.p.) area under the curve measurements of the CBF response showed no significant differences to those obtained from control animals (Fig. 4C). Given that the BOLD signal measurements described above were acquired with a temporal resolution of 120 s, an integrated measure of total CBF response over time was used to characterise the stimulus evoked CBF signal change rather than a readout of the transient response maxima.

### Effect of 5-HT_2A_ receptor blockade on fenfluramine-induced BOLD responses

**Fig. 5:**
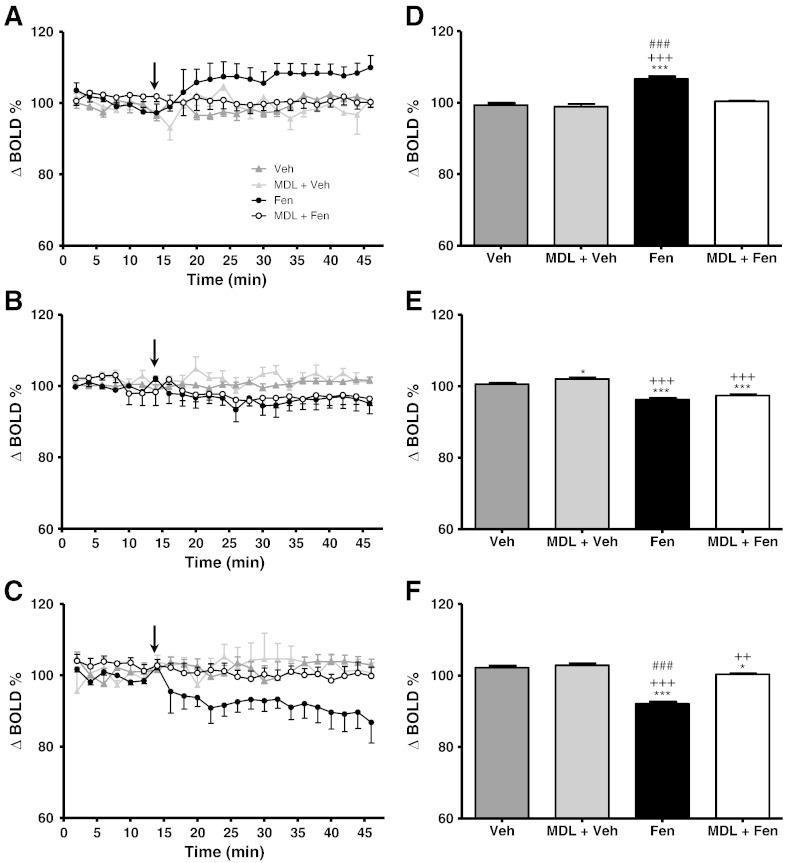
(A–C) Time course plots showing the change in BOLD signal intensity in (A) motor cortex, (B) nucleus accumbens and (C) dorsal raphe nucleus following i.v. administration of saline or fenfluramine (10 mg/kg). In two groups animals were pre-treated with the selective 5-HT_2A_ receptor antagonist MDL100907 (0.5 mg/kg i.p.) 15 min prior to i.v. fenfluramine (MDL-fen) or vehicle (MDL-veh) injection. Data represent mean ± SEM (n = 5 per group). Fenfluramine or vehicle was administered i.v. at t = 15 min on the time course plots (arrow; MDL100907 was administered at t = 0 min relative to these plots). (D–F) Bar graphs showing mean ± SEM of the signal intensity over the first 30 min after fenfluramine or saline administration. *p < 0.05, ***p < 0.0001 (with respect to Veh-Veh); ^++^p < 0.01, ^+++^p < 0.0001 (with respect to MDL-Veh); ###p < 0.0001 (with respect to MDL-Fen).

Our previous studies demonstrated that fenfluramine-evoked changes in activity-dependent gene expression involved 5-HT_2A_-receptor activation (Hirani et al., 2003). For the time course analysis (15 min baseline + 31 min post-fenfluramine or vehicle) pre-treatment with the selective 5-HT_2A_ receptor antagonist MDL100907 significantly attenuated the BOLD response to fenfluramine compared to untreated animals in the MCx and DRN (ANOVA group F_3,14_ = 22.31, time F_7,460_ = 38.79, group:time F_21,460_ = 38.79; post-hoc MCx p < 0.0001, DRN; p < 0.0001; Figs. 5A, C). In these regions both groups pre-treated with MDL (MDL-Fen and MDL-Veh) were significantly different to the animals given fenfluramine alone (*post-hoc* Tukey p < 0.0001), but not to the control animals given vehicle alone. In contrast, in the NAc MDL100907 did not appear to ameliorate the response to fenfluramine, with both MDL-Fen and fenfluramine alone groups showing significant differences to the MDL-Veh and vehicle alone groups (*post-hoc* Tukey p < 0.0001; Fig. 5B), but not to each other.

Similarly, in the MCx and DRN, analysis of the first 30 min post-fenfluramine (or vehicle) administration showed that the response to fenfluramine was largely abolished with MDL100907 pre-treatment (Fig. 5D, ANOVA group p < 0.0001 F_3,14_ = 39.33; *post-hoc* Tukey p < 0.001). No significant differences were found between the MDL-Fen, MDL-Veh and vehicle alone groups in the MCx (Fig. 5D). However, although only minor differences in the BOLD response time course were evident between the MDL-Fen, MDL-Veh and vehicle only groups in the DRN (Fig. 5C), these differences reached significance in the analysis of the first 30 min post-fenfluramine (*post-hoc* Tukey p < 0.01; Fig. 5F) suggesting some residual effect of fenfluramine. As for the time course analysis, no significant difference in the BOLD response of the NAc to fenfluramine was seen in the MDL-Fen group compared to the fenfluramine alone group in the analysis of the first 30 min post-fenfluramine injection (Fig. 5E). At the same time, the MDL-Fen group showed significant differences to both the vehicle only and MDL-Veh groups (*post-hoc* Tukey, p < 0.05 and 0.001, respectively; Fig. 5E) confirming minimal effects of 5-HT_2A_ antagonism on the BOLD response to fenfluramine in the NAc.

To confirm that MDL100907 pre-treatment did not alter the baseline BOLD signal the initial 15 min of baseline in the two MDL100907 treated groups was compared with the 15 min of baseline acquisition post-MDL100907 administration. No significant differences were found.

### Systemic inflammation and 5-HT_2A_ receptor expression

**Fig. 6:**
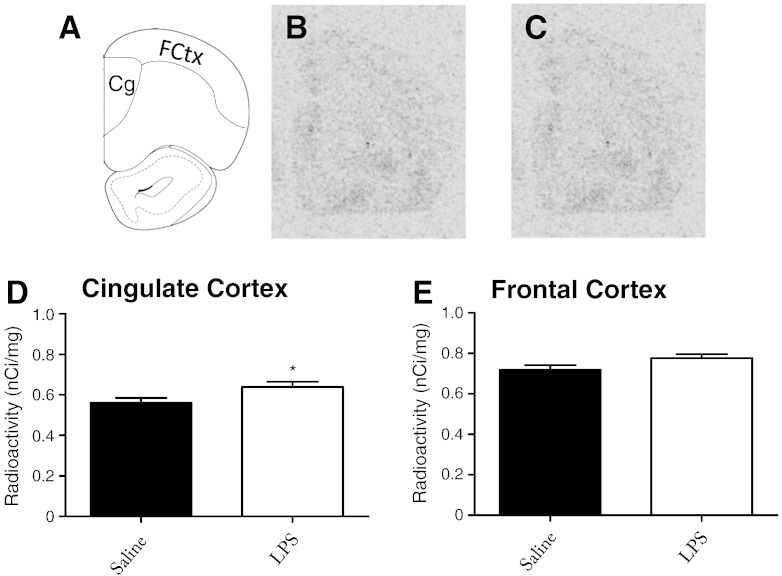
5-HT_2A_ protein expression in the rat brain 6 h after an LPS challenge. Animals received a single i.p. dose of LPS 6 h prior to sacrifice, brains were sectioned coronally and frontal regions were subjected to ^3^H-ketanserin autoradiography. The cingulate MCx and frontal MCx were defined (A) and analysed using MCID Core Software. Representative photomicrographs from film autoradiograms from (B) saline and (C) LPS animals. Analysis shows data from (D) cingulate MCx and (E) frontal MCx. Data are mean ± SEM (n = 3), *p < 0.05.

Changes in BOLD responses after systemic inflammation and in response to 5-HT_2A_ drugs may reflect post-synaptic changes in receptor expression. Tissue was cryosectioned for autoradiographic analysis using ^3^H-ketanserin, a 5-HT_2A_ ligand. Expression of the 5-HT_2A_ receptor is largely restricted to the prefrontal areas of the brain, therefore cryosections did not extend beyond the start of the hippocampus (Lopez-Gimenez et al., 1997). 5-HT_2A_ expression was measured in saline (Fig. 6B) and LPS (Fig. 6C) brains and was found to be significantly increased in the cingulate MCx (Student’s t-test; p < 0.05) and elevated, but not significantly different from saline challenged animals (p = 0.06), in the frontal MCx.

### Effects of LPS on 5-HT_2A_-mediated behaviour

**Fig. 7:**
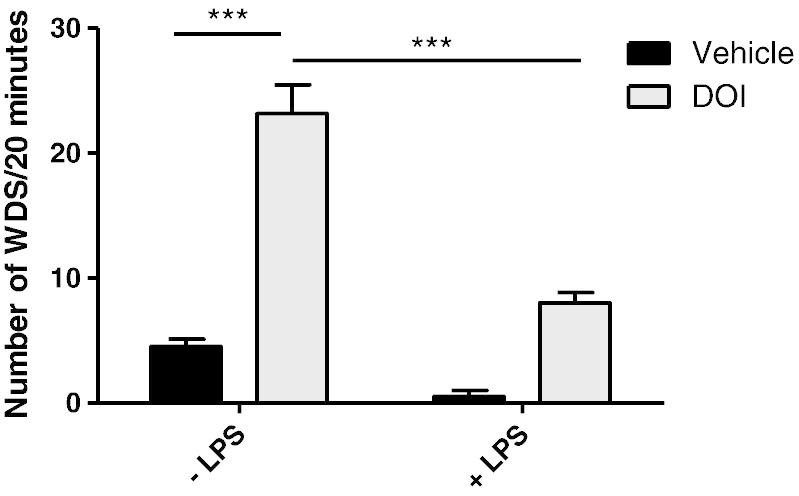
LPS-induced inhibition of wet dog shake (WDS) behaviour in rats. Animals received a single i.p. dose of LPS (0.5 mg/kg) or saline and were allowed to recover for 6 h prior to a single subcutaneous injection of the 5-HT_2A_ agonist DOI (1 mg/kg) or vehicle (saline). WDS behaviour was evaluated over a 20-minute period. Data represent mean ± SEM (n = 6/group), ***p < 0.001.

Whilst changes in 5-HT_2A_ receptor expression and the administration MDL100907 strongly indicate that LPS might alter 5-HT_2A_ signalling, it was important to demonstrate functional effects in a 5-HT_2A_-dependent behaviour. With this in mind, we used a direct 5-HT_2A_ agonist (±)-1-(2,5-dimethoxy-4-iodophenyl)-2-aminopropane (DOI; 1 mg/kg subcutaneous) in the presence and absence of systemic inflammation. Subcutaneous DOI administration in laboratory animals elicits a very specific behavioural response; in rats it is known as wet dog shake (WDS) behaviour. Both LPS and DOI had a significant effect on WDS shake behaviour, there was also a significant interaction (two-way ANOVA; DOI p < 0.001 F_1,19_ = 124.3; LPS p < 0.001 F_1,19_ = 38.17; LPS:DOI p < 0.001; F_1,19_ = 29.31). Post-hoc analysis shows that when compared to vehicle, DOI induced significantly more WDS 6 h after a 0.1 ml i.p. injection of 0.9% saline (Bonferroni *post-hoc*; p < 0.001). Administration of a single dose of 0.1 ml LPS in saline (0.5 mg/kg i.p.) 6 h prior to DOI significantly reduced the number of observable WDS compared to the saline pre-treated control animals (Bonferroni *post-hoc* p < 0.001 Fig. 7).

## Discussion

The principal findings of the present study are that phMRI-measured brain responses to the 5-HT releasing agent fenfluramine were markedly reduced by prior administration of the systemic inflammatory agent LPS, and that mechanistically these effects may be mediated by changes in post-synaptic receptor expression. Our data indicate not only that systemic inflammatory pathways affect central 5-HT function, but that functional MRI outcomes, used in both animals and man, can be altered by systemic immune system activation. Our parallel microdialysis experiments showed that LPS did not directly reduce fenfluramine-evoked 5-HT release, thereby ruling out a presynaptic 5-HT mechanism in this instance. Direct recordings of stimulus evoked blood flow responses further show that the effects of LPS on the fMRI response to fenfluramine were not due to disruption of vascular reactivity or neurovascular coupling mechanisms. Finally, results using 5-HT_2A_-targeting drugs indicate that some aspects of sickness behaviour may be mediated by the 5-HT_2A_ receptor, whilst the fenfluramine evoked BOLD signal changes were attenuated by pre-treatment with the selective 5-HT_2A_ receptor antagonist, MDL100907. Taken together, these data suggest that contrary to predictions from previous studies, the inhibitory effect of systemic inflammation on central 5-HT function is mediated by a post- and not presynaptic 5-HT mechanism, and that a downstream reduction in 5-HT_2A_ receptor signalling may play a key role in this inhibition.

### Decreased BOLD responses to fenfluramine after systemic inflammation

We have recently reported that the 5-HT releasing agent fenfluramine elicits a pattern of BOLD signal changes across several brain regions of the anaesthetised rat. This effect was attenuated by prior 5-HT depletion, suggesting that the BOLD response to fenfluramine is a surrogate marker of increased 5-HT release (Preece et al., 2009). Consistent with these data, human studies report that the selective 5-HT re-uptake inhibitor citalopram, also evokes BOLD responses (McKie et al., 2005). As shown previously, fenfluramine evoked region-specific changes in BOLD signal in the current study. Pre-treatment with LPS markedly attenuated the BOLD responses to fenfluramine. This finding is in accord with human fMRI studies, which show that changes in neuronal activity may be altered by states of heightened immune compromise (Brydon et al., 2008). Given the evidence that the fenfluramine-evoked BOLD changes are 5-HT-dependent (Preece et al., 2009), it seems likely that the effect of LPS on the BOLD responses to fenfluramine is also mediated by alteration of central 5-HT function.

Importantly, cortical *in vivo* microdialysis experiments demonstrated that LPS administration did not alter either basal extracellular 5-HT levels or its metabolite 5-HIAA, nor fenfluramine-evoked release of cortical 5-HT. In previous studies we have demonstrated that fenfluramine not only evoked cortical 5-HT release similar in magnitude to that observed here, but that the 5-HT response to fenfluramine was markedly attenuated in animals with depleted brain levels of 5-HT (Series et al., 1994). Thus, in the MCx, at least, decreased 5-HT release does not appear to underlie the LPS-induced suppression of BOLD responses to fenfluramine.

Since systemic inflammation is well known to affect the vasculature (Brydon et al., 2008), it was important to test whether LPS treatment disrupted mechanisms linking changes in neuronal activity to the hemodynamic responses. To this end, a model that we have previously used to study neurovascular coupling (Martin et al., 2006) was used to verify that functional hyperaemic responses remained intact in LPS treated animals. Although there was some variation in responses between LPS- and saline-treated animals, these experiments demonstrated that it was still possible to elicit robust haemodynamic responses to increased neuronal activity in the LPS treated animals. Previous studies have shown that endotoxin-induced inflammation significantly alters CBF and disrupts autoregulation (Rosengarten et al., 2008a, 2008b). Moreover it has been hypothesised that systemic endotoxin causes a direct uncoupling of the cerebral microvasculature from its neuronal input, but that this is not caused by inflammation-induced oedema (Rosengarten et al., 2008a, 2008b). In contrast, the current data show that in the MCx, at least, neurovascular coupling mechanisms remain intact during the acute phase of systemic inflammation. In the current study, a lower LPS dose was used (0.5 mg/kg *vs*. 1–5 mg/kg) than that of Rosengarten et al. and this may explain the discrepancy with earlier work.

### BOLD responses to fenfluramine: role of 5-HT_2A_ receptors

As shown previously (Preece et al., 2009), fenfluramine induced a positive BOLD response in the MCx, but negative responses in both the DRN and NAc. Pre-treatment with the selective 5-HT_2A_ antagonist MDL100907 effectively blocked the BOLD response to fenfluramine in both MCx and DRN, whilst attenuation of the BOLD response in the NAc was only partial. These findings suggest a strong involvement of the 5-HT_2A_ receptor in the BOLD response to increased 5-HT in the MCx and DRN, but the involvement of additional receptor(s) for 5-HT or other neurotransmitters in the NAc. Candidate excitatory 5-HT receptor subtypes in NAc that would not be blocked by MDL100907 include 5-HT_2C_, 5-HT_4_ and 5-HT_6_ receptors. Previous studies have detected non-5-HT-mediated changes in Fos response to fenfluramine in the caudate nucleus (Javed et al., 1998), and other studies report that fenfluramine releases catecholamines at higher doses (Balcioglu and Wurtman, 1998). Thus, although fenfluramine may primarily release 5-HT, a minor contribution from other transmitters is possible in the NAc.

The BOLD increase in MCx induced by fenfluramine most likely reflects activation of postsynaptic 5-HT_2A_ receptors, which are excitatory and abundant in this region. In comparison, there is a low abundance of 5-HT_2A_ receptors in the DRN suggesting that a component of this effect may be mediated by 5-HT_2A_ receptors located outside the DRN. We have previously demonstrated evidence for a feedback loop from the anterior MCx to the DRN involving cortical glutamatergic neurones that synapse onto local GABAergic neurons in the DRN (Hajos et al., 1998; Varga et al., 2001), which is likely mediated by 5-HT_2A_ receptors in MCx (Boothman et al., 2003; Sharp et al., 2007). This cortical-raphe control system can be altered under conditions of stress has been reviewed by Stein et al. (2007). Thus, the fenfluramine-evoked decrease in BOLD signal observed in the DRN might, in part, be triggered by cortical 5-HT_2A_ receptors, which excite cortico-raphe projections leading to a reduction in neuronal activity in the DRN through activated local GABAergic neurons. The fenfluramine-evoked BOLD decrease observed in NAc may reflect activation of local 5-HT_2A_ receptors on inhibitory GABA neurons (Ward and Dorsa, 1996), although there is likely a contribution from additional mechanisms as discussed above.

It should be noted that the BOLD responses measured herein may not be entirely neurally mediated. There is much evidence to suggest that certain 5-HT receptors, including 5-HT_2A_ receptors, are expressed on the microvasculature of the CNS (Brydon et al., 2008). Whilst the cellular location and function of these receptors remains unclear, there is evidence to suggest that 5-HT_2A_ receptors mediate both vessel contraction (Martin et al., 2006) and dilation (Rosengarten et al., 2008a, 2008b) in the brain. Although there is a report suggesting that 5-HT_2A_ receptor blockade inhibits CBF in a model of cortical spreading depression (Gold et al., 1998), it was noted that factors such as vascular size and presence of the blood brain barrier may affect whether 5-HT dilates or contracts arterioles. Nevertheless, some of the observed effects of 5-HT_2A_ blockade may reflect modulation of these directly vascular actions of 5-HT on 5-HT_2A_ receptors.

### 5-HT_2A_ receptors in sickness behaviour and depression

There is much evidence that links 5-HT_2A_ receptors to depression and a decrease in 5-HT_2A_ receptor function would seem in keeping with the general view that decreased 5-HT function is an important depression vulnerability factor. However, data regarding the role of 5-HT_2A_ receptors in depression are currently contradictory. For example, some post-mortem studies of depressed suicide victims report decreased 5-HT_2A_ receptor binding^23^, whilst others show an increase^21-22^. Positron emission tomography studies have also investigated 5-HT_2A_ receptor expression in depressed patients, but findings to date have been inconsistent (Bhagwagar et al., 2004; Meyer et al., 1999). On the other hand, 5-HT_2A_ agonists are being pursued as a target to relieve depression and to trigger molecular effects linked to antidepressant action. Oddly, 5-HT_2A_ antagonists also have antidepressant augmenting properties (Celada et al., 2004). Thus, the relationship between systemic inflammation and changes in 5-HT function observed here cannot yet be readily linked to depression vulnerability. In our study, expression of the 5-HT_2A_ receptor in the prefrontal MCx of animals increased in response to systemic inflammation. Other studies using different inflammatory agents such as carrageenan have also shown increased 5-HT_2A_ receptor expression (Zhang et al., 2001). Non-inflammatory stressors also cause an increase in 5-HT_2A_ receptor expression in the MCx and in the hippocampus (Watanabe et al., 1993). The precise mechanism of activation of increased 5-HT_2A_ receptor expression is unclear, but it may involve direct cytokine induced gene expression changes, but it may also reflect indirect mechanisms. In culture, cytokines have been shown to reduce signal transduction through the 5-HT_2A_ receptor, *via* mechanisms involving subcellular kinases (Kugaya et al., 1995). Cortisol and corticotrophin releasing hormone are induced by circulating cytokines, and it is also possible that the 5-HT_2A_ expression changes may also be a consequence of HPA axis activation (Leonard, 2005). HPA axis activation appears to be a homeostatic response to elevated cytokine expression and it is often difficult to work out whether it is the afferent or efferent arm of the CNS response that is responsible for the molecular changes associated with injury or disease. Our BOLD data and the expression data provide good evidence to infer a direct effect of LPS on 5-HT_2A_ receptor signal transduction, but the demonstration of a direct action of LPS on 5-HT_2A_ signalling was important. The DOI data reported here demonstrate that LPS administration does result in a significant loss of 5-HT_2A_-mediated behaviour despite increased receptor expression. It is not unusual for mRNA levels and protein levels to increase, as the functionality is lost in many biological scenarios. For example, chronic fluoxetine treatment increases 5-HT_2A_ receptor binding potential in the frontal cortex, whilst fenfluramine-induced prolactin release was blunted, suggesting desensitisation of the 5-HT_2A_ receptor (Sawyer et al., 2012). Interestingly, in their study, measures of pre-synaptic serotonergic function were unaffected. These results extend the work of Kouhata et al. (2001) who showed that at 1 and 3 h after LPS, DOI-induced WDS were decreased in a cyclooxygenase-dependent manner. The 0.5 mg/kg dose of LPS used in this experiment, and in those of Kouhata, did inhibit spontaneous locomotor activity and might be viewed as a confound in the WDS paradigm. However, data from other studies, using pro-inflammogens at concentrations which do not inhibit locomotor activity, still show a reduction in WDS behaviour (Kugaya et al., 1996) suggesting that the behaviour is 5-HT_2A_ specific.

In summary, the current data suggest that the mechanisms underlying the effects of systemic inflammation on the BOLD response to fenfluramine are not pre-synaptic in origin, specifically through compromised 5-HT release, and that they do not reflect disruption of neurovascular coupling capacity. However, the pattern of LPS-induced effects is strikingly similar to those observed with a specific 5-HT_2A_ receptor antagonist. These findings, in combination with inflammation-induced changes in 5-HT_2A_ expression and 5-HT_2A_-mediated behaviours, suggest that the effects of systemic inflammation on central 5-HT function reflect, at least in part, modulation of 5-HT_2A_ signalling pathways.
