# Corrigendum to “Systemic inflammation alters central 5-HT function as determined by pharmacological MRI” [Neuroimage 75 (2013) 177–186]

**DOI:** 10.1016/j.neuroimage.2014.03.021

**Published:** 2014-07-01

**Authors:** Yvonne Couch, Chris J. Martin, Clare Howarth, Josie Raley, Alexandre A. Khrapitchev, Michael Stratford, Trevor Sharp, Nicola R. Sibson, Daniel C. Anthony

**Affiliations:** a Department of Pharmacology, University of Oxford, Mansfield Rd., Oxford OX1 3QT, UK; b CR-UK/MRC Gray Institute for Radiation Oncology and Biology, Department of Oncology, University of Oxford, Churchill Hospital, Oxford OX3 7LJ, UK

The authors wish to add the following statement to the Acknowledgments section of their article on page 185:

This work was funded by the MRC UK (grant number G0401355), a BBSRC UK studentship (Y.C.) and the Wellcome Trust (grant number WT085523MA).

